# The Tuberculosis Society

**Published:** 1914-06-06

**Authors:** 


### The Hospital

**The Workers' Newspaper of Administrative Medicine and Institutional Life, Administration, National Insurance and Health.**

No. 1458, Vol. LVI. SATURDAY, JUNE 6, 1914. PRICE ONE PENNY.

### THE TUBERCULOSIS SOCIETY.

For some time there has been evident a wish, on the part of tuberculosis officers and others exclusively concerned with this disease, for some kind of professional union. The correspondence columns of the medical journals have contained many short letters broaching the subject, letters falling into the inevitable three classes. The first consisted in those expressing much what has been said in our opening sentence—the sentiment, that is, of solidarity; and while, of course, the most important of all, in a way, as proving the prevalence of the desire, did not go much into particularities and so need not detain us. In the second, fewer in numbers, appeared the proposal that tuberculosis officers (who must, of course, form the bulk of membership of any such society) would find their interests best served by making their association a branch of the fairly well-known Society of Medical Officers of Health. A precedent was given, that of the education medical officers, who had taken this course, and it was pointed out that besides clinical matters there was a good deal of a material kind to be done to improve tuberculosis officers' prospects, in the way of security of appointment tenure, of prospective pension, and the like. Last came a single communication, from the secretary of the body named in the title of these remarks, mentioning that his society was in its second year, had over sixty members and Professor Osler as an honorary president, and that he would be glad to hear from all concerned who—and so on. Clearly, then, this gentleman's letter, which, by the way, is fully verified by a printed prospectus, has to be weighed against those mentioned just before it.

Now that not very ideally minded person, the Aristotelian common-sense man—" apt to do "—would be in no doubt at all about the matter. Possession is nine points of the law, he would say: those who have actually taken the thing in hand are the ones to be encouraged. And while not subscribing largely to the mournfully often true adage he cites, we immediately agree that the energy which has been shown deserves support and backing. Primarily, however, not from utilitarian motives, not because it would be a pity to see a small and junior body of medical men split up into two camps, but because the basis of the Association seems the only sound one, being indeed clinical efficiency. Article 2 runs: " Object. The society shall be for the study and discussion of all subjects relating to the control and eradication of tuberculosis." If we compare this primary object with the more selfish ones set forth above, the Tuberculosis Society comes well out of the comparison. All the more because, indeed, as a whole, tuberculosis officers have as yet no great efficiency to boast of. The mistake has been freely made by election committees of laying the stress upon the administrative side of the work. A recent advertisement postulates at least three months spent at a dispensary, but says nothing of any other place where special experience may be got. This, of course, is ridiculous. A great part of the work lies in picking out early disease, the requisite diagnostic ability for which can only be acquired, like age, " with stealing steps." On the other hand, any person of ordinary ability can pick up office work and insurance rules in a week. And if one faces the question of self-interest—necessary, after all, to consider in a workaday world—would this really be better furthered by an ancillary institution than by the Tuberculosis Society? Hardly, perhaps, for many whole-time medical officers of health are, and have long been, quite at the mercy of a smallish municipal body's sense of fairness, while scarcely any of them can look forward to pensions. Their society, no doubt, has its eye on these points and a good notion of the best way to set about getting them; but, naturally enough, it will be its original members it will work for first of all. Again, a sanitarian can never be much of a clinician; and the tuberculosis officer's duties, unlike those of the education officer, are very largely clinical. And if the medical officer of health, who, with the increase of work late years have brought him (not, be it observed, from any research on his own part), can hardly be very *eingehend* in his knowledge of tuberculosis, were at some future date to be relieved a little—if there were mooted a "hiving off"—would not an independent organisation show better in support of the proposal than a dependent one? Such speculations, such, as the phrase goes, legitimate ambitions, will no doubt have occurred to many of those now engaged in tuberculosis work under the Insurance Act, whether in dispensary or in sanatorium.

In fine, a sense both of the general weal—of the right future for sociological medicine—and of the particular well-being of whole-time tuberculosis workers inclines us to advise every possible candidate for membership, who has not already done so, to send five shillings to Dr. W. O. Pitt, Walthamstow, Essex. His society might also be advised (again, if it have not already taken steps) to establish relations with the kindred bodies in Ireland and in Germany; and by all means to announce prominently its meetings and the agenda. But we are quite prepared to consider other points of view, and to open our columns to a discussion of the subject.

